# Effect of Danshen for improving clinical outcomes in patients with bladder cancer: a retrospective, population-based study

**DOI:** 10.3389/fphar.2023.1260683

**Published:** 2023-12-11

**Authors:** Yi-Hsin Chen, Chih-Tsung Chen, Han-Ping Wu

**Affiliations:** ^1^ Department of Nephrology, Taichung Tzu Chi Hospital, Taichung, Taiwan; ^2^ School of Medicine, Tzu Chi University, Hualien, Taiwan; ^3^ Department of Artificial Intelligence and Data Science, National Chung Hsing University, Taichung, Taiwan; ^4^ College of Medicine, Chang Gung University, Taoyuan, Taiwan; ^5^ Department of Pediatrics, Chiayi Chang Gung Memorial Hospital, Chiayi, Taiwan

**Keywords:** Danshen therapy, bladder cancer, cardiovascular outcomes, traditional Chinese medicine, TCM

## Abstract

**Introduction:** Traditional Chinese Medicine (TCM) has a broad application in healthcare, with Danshen being a notable herb used in Eastern medicine for cancer treatment. This study aims to explore the relationship between Danshen use and cardiovascular risks among bladder cancer patients.

**Methods:** Patients were selected based on a confirmed diagnosis of bladder cancer with specific inclusion and exclusion criteria to control for certain comorbidities and treatments. Utilizing Taiwan’s National Health Insurance data from 2003 to 2013, this retrospective, population-based study identified three groups: 525 patients treated with Danshen, 6,419 patients not treated with TCM, and 4,356 patients treated with TCM but not with Danshen. The Cox proportional hazard model was employed to estimate the risks of Major Adverse Cardiovascular Events (MACE) and mortality while accounting for various confounders.

**Results:** The overall incidence of MACEs was significantly lower in the Danshen group (5%) compared to the TCM (8.1%) and non-TCM (9.9%) groups (*p* < 0.001). The Cox model revealed that bladder cancer patients treated with Danshen had the lowest risk of MACE (adjusted hazard ratio, 0.56; 95% confidence interval, 0.38–0.84) and all-cause mortality (adjusted hazard ratio, 0.60; 95% confidence interval, 0.44–0.82).

**Discussion:** The findings suggest that Danshen reduces the risk of MACE and all-cause mortality in bladder cancer patients, highlighting its potential benefits. This underpins the necessity for further research to substantiate the cardiovascular benefits of Danshen in bladder cancer patients and potentially broaden its application in oncology healthcare.

## 1 Introduction

Bladder cancer is one of the 10 most prevalent malignancies worldwide, with approximately 550,000 new cases diagnosed annually (Richters et al., 2020). Although only 5% of patients initially have metastatic bladder cancer, many patients treated for localized illness relapse or develop advanced stages, with a 5-year relative survival rate of 4.6% (Flaig et al., 2020). Cisplatin-based chemotherapy, which often causes nephrotoxicity, remains the standard treatment for eligible patients with bladder cancer, despite the emergence of immunotherapy (Flaig et al., 2020).

The use of traditional Chinese medicine (TCM) in healthcare is widespread in many Asian and Western countries. Owing to the quality of finished herbal products and their ease of use, TCM doctors often prescribe contemporary forms of decoctions, in which herbal formulas and single herbs are condensed in granulated compounds. Taiwan’s National Health Insurance (NHI) program pays claims for finished herbal products, including individual herbs and formulas. As both clinical pharmaceuticals and TCM are often used to explore their clinical effects on patients in Taiwan, the NHI Research Database (NHIRD) of Taiwan provides a wealth of information about the prescriptions, procedures, and diagnoses of patients (Chang et al., 2015; Fleischer et al., 2017; Hung et al., 2017; Lin et al., 2017).

The association between cancer and excess risk of heart disease has received particular attention in recent years (Cardinale et al., 2008; Stoltzfus et al., 2011; Herrmann et al., 2014). Although the mechanism underlying this link is complex, both conditions are speculated to share similar pathophysiological pathways of chronic inflammation and oxidative stress (Johnson et al., 2016). The cardiovascular risk in patients with cancer is further increased by the possible cardiotoxicity associated with cancer therapy. Thus, healthcare professionals are now aware of the common risk factors and are paying specific attention to the cardiovascular health of oncology patients receiving cardiotoxic anticancer medications.

Danshen, derived from the dried root of Salvia miltiorrhiza Bunge, is a traditional Chinese medicine (TCM) agent used predominantly for various endocrine and cardiovascular ailments, encompassing menstrual irregularities, hepatitis, and coronary artery disease. In the context of this study, Danshen was employed as a singular agent. Its bioactive components, notably TSN IIA, have been recognized for their cardioprotective and potential anti-cancer attributes. The influence of Danshen on the progression or therapeutic approach to bladder cancer remains an active area of scholarly investigation. (Han et al., 2002; Liu et al., 2016; Wang et al., 2017; Li et al., 2018; Yuan et al., 2019). Danshen improved the biochemical indicators in 126 patients at a 3-month follow-up, demonstrating a decreased risk of coronary heart disease (Liu et al., 2016). In addition to improving the ejection fraction and fractional shortening, Danshen injections also improved heart function. In a mouse model of heart failure caused by ligation of the left anterior descending coronary artery, intramuscular Danshen injections at a dose of 1.5 mL/kg/d for 14 days prevented left ventricular remodeling (Wang et al., 2017). Therefore, this study aimed to analyze the effects of Danshen on survival and major adverse cardiac events (MACE) in patients with bladder cancer.

## 2 Materials and methods

### 2.1 Ethics

This study was approved by the Institutional Review Board of Buddhist Taichung Tzu Chi General Hospital in Taiwan (REC111-14) and was conducted in accordance with the tenets of the Declaration of Helsinki. Before the analysis, the NHIRD identification numbers of individuals were removed from the dataset. The review board waived the requirement for patient consent because of the retrospective nature of the study.

### 2.2 NHI Research Database

The NHI, a single-payer system managed by the government, was established in Taiwan in 1995. Currently, 99% of Taiwanese residents participate in the NHI program (Chiang, 1997). The National Health Research Institutes collect data related to these services and input them into the NHIRD. It includes inpatient records, outpatient records, medication records, and registration files. The National Health Research Institutes of Taiwan released NHIRD data from 1 January 2003, to 31 December 2013, which were utilized in this investigation. The International Classification of Diseases, Ninth Revision (ICD-9) codes were used in this study, along with records of admissions and outpatient visits, which included patient characteristics such as sex, date of birth, date of admission, date of discharge, date of visit, and up to three diagnoses made during an outpatient visit. The names of the prescribed medications (both Western medicine and TCM), their dosages, durations, total costs, and additional treatments were obtained from the patient prescriptions for this study.

### 2.3 Danshen exposure and control group selection

**FIGURE 1:**
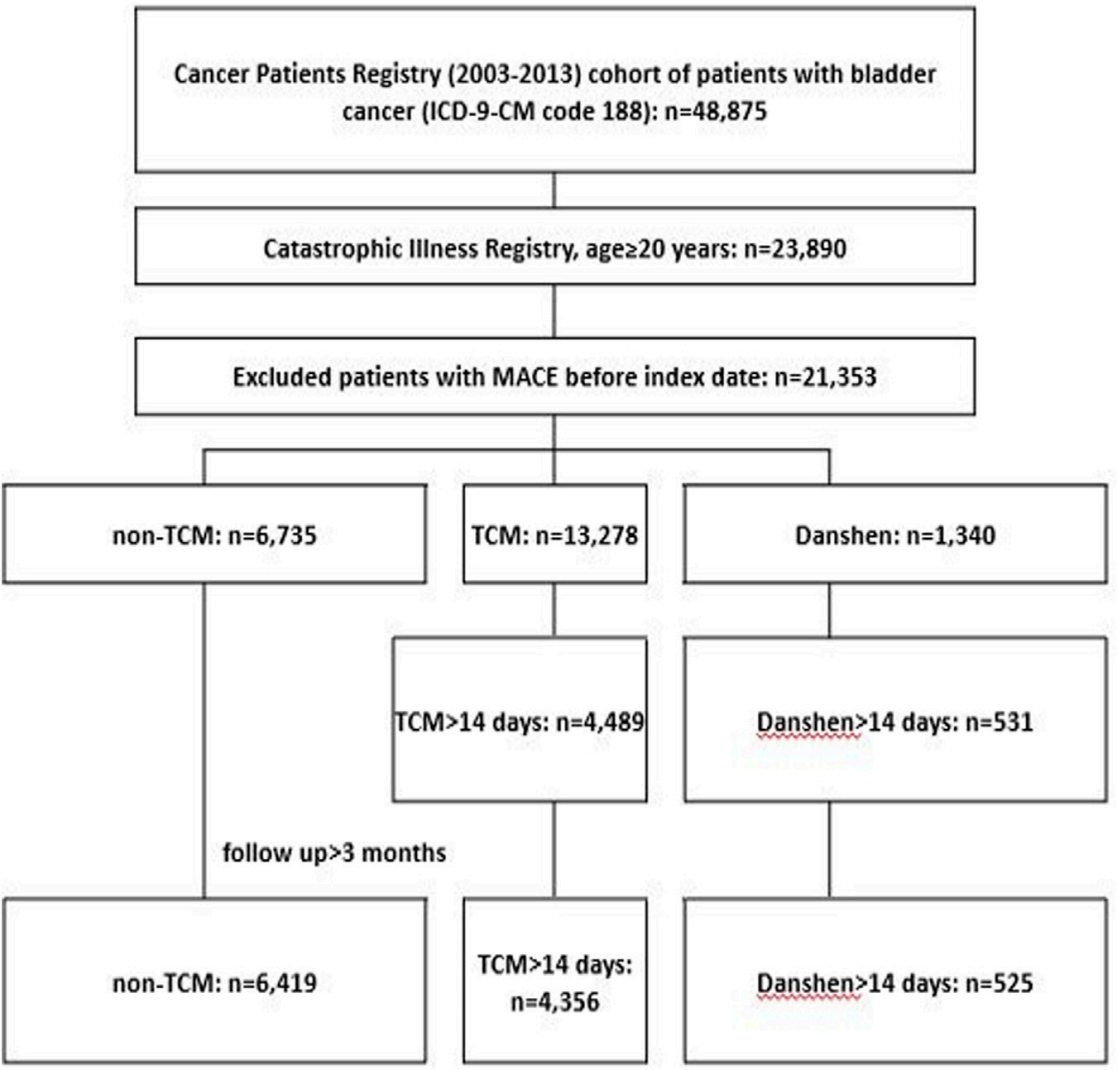
Study design flow chart. Danshen, patients who used Danshen; ICD-9-CM, International Classification of Diseases, ninth revision; MACE, major adverse cardiac events; non-TCM, patients who did not use any traditional Chinese medicine; TCM, patients who used traditional Chinese medicine other than Danshen.

The participants were selected from the Registry for Catastrophic Illness Patients of the NHIRD (Figure 1). Patients with bladder cancer were considered eligible if the diagnosis was registered in the Catastrophic Illness database. First, we included patients newly diagnosed with bladder cancer (ICD-9-CM 188) between 1 January 2003, and 31 December 2013 (*n* = 48,873). Individuals utilizing Danshen for a duration exceeding 30 days, while adhering to a typical clinical dosage of 3 g per day—a standard in clinical Chinese medicine practices, referenced from (Lin et al., 2019) and who received a bladder cancer diagnosis (confirmed through the application for the cancer catastrophic illness certificate) were categorized as Danshen users. In contrast, those employing TCM without incorporating Danshen were identified as TCM users, while those who underwent treatment for a period less than 30 days or abstained from any TCM were classified as non-TCM users. We excluded patients with a follow-up of <3 months and those with MACE that occurred before the index date. The presence of at least three outpatient visits by the same person was confirmed to support these diagnoses. Figure 1 illustrates the inclusion of a matched cohort of 11,300 individuals from an initial sample of 48,875 patients, based on these criteria. From 1 January 2003, to the date of death, date of removal from the registry, or 31 December 2013, the enrolled patients accrued the follow-up time.

### 2.4 Covariates

The sociodemographic factors included age and sex. Baseline comorbidities were considered if the following ICD-9-CM codes appeared at least once in patients or inpatients before the initial bladder cancer diagnosis: diabetes mellitus (ICD-9-CM: 250) and hypertension (ICD-9-CM: 401–405). Charlson Comorbidity Index Score (CCIS)-related diagnoses were considered in the analysis, including myocardial infarction (ICD-9-CM: 410, 412), congestive heart failure (ICD-9-CM: 42, 785.4), peripheral vascular disease (ICD-9-CM: 44x), cerebrovascular disease (ICD-9-CM: 43x), dementia (ICD-9-CM: 290), chronic obstructive pulmonary disease (ICD-9-CM: 49x, 500–506), connective tissue disease (ICD-9-CM: 710.x), peptic ulcer disease (ICD-9-CM: 533.xx), chronic kidney disease (ICD-9-CM: 582, 583, 585, 586, 588), hemiplegia (ICD-9-CM: 342, 344.1), leukemia (ICD-9-CM: 204, 206, 207, 208), malignant lymphoma (ICD-9-CM: 200, 201, 202, 203, 205), solid tumor (ICD-9-CM: 196–199), and liver disease (ICD-9-CM: 571.2, 571.4, 571.5, 571.6).

### 2.5 Statistical analysis

SAS 9.4 (SAS Institute Inc., Cary, NC, United States) was used for all analyses. Baseline attributes are reported as number and percentage for categorical data and as mean and standard deviation for continuous data. For categorical data, the χ2 and Fisher’s exact tests were used. Analysis of variance was used for continuous variables. The threshold for statistical significance was set at a confidence level of alpha = 0.05. The life table technique (SAS LIFETEST program) was used to estimate the Kaplan–Meier survival curves for all-cause mortality and MACE, and the log-rank test was used to analyze the differences among the Danshen, TCM, and non-TCM groups. Sex, diabetes, hypertension, myocardial infarction, congestive heart failure, peripheral vascular disease, dementia, connective tissue disease, peptic ulcer disease, chronic kidney disease, hemiplegia, leukemia, malignant lymphoma, solid tumors, and liver disease were adjusted for in all Cox proportional hazard models.

## 3 Results

### 3.1 Patient characteristics

A total of 4,881 patients (3,389 males and 1,492 females) with bladder cancer who were prescribed Chinese medicine in Taiwan were included in our study cohort, which was obtained from the NHIRD. Another 6,419 patients with bladder cancer who did not use Chinese medicine were included in the control group, consisting of 5,128 male and 1,291 female patients. Among the student population, we identified 525 patients treated with Danshen, 4,356 patients treated with TCM but not Danshen, and 6,419 patients not treated with TCM. A comparison of the demographic characteristics, CCIS-related comorbidities, MACE, mortality rates, length of stay in the first hospitalization, socioeconomic status, and geographic regions among patients in the stress groups is shown in Table 1. Patients in the non-TCM group were significantly older than those in the other two groups (*p* < 0.001). CCISs of patients in the Danshen and non-TCM groups were higher than those of patients in the TCM group (*p* < 0.05). The proportion of myocardial infarction was not significantly different among the three groups, although the Danshen group had the highest proportion (11.2%), followed by the non-TCM group (10.6%) and the TCM group (8.8%). For cerebrovascular diseases, the non-TCM group had the highest proportion (5.0%) compared with the other two groups (3.7% for TCM and 2.7% for Danshen) (*p* < 0.01). The frequency of hypertension was higher in the non-TCM group (45.0%) than in the TCM (41.0%) and Danshen (42.3%) groups (*p* < 0.001). In addition, the Danshen group had the lowest MACE rate during the short-term follow-up compared with the TCM and non-TCM groups (*p* < 0.001). The Danshen group had the lowest overall mortality rate (8.0%), whereas the non-TCM group had the highest mortality rate (14.6%). During the first hospitalization, the TCM group had the lowest rate (68.1%), followed by the Danshen group (70.1%). During the first hospitalization, the patients in the Danshen group had the longest length of stay (9.9 months) compared with those in the non-TCM (2.7 months) and TCM (6.5 months) groups. No significant differences were noted between patients from different geographic regions.

**TABLE 1:** Comparison of demographic characteristics among patients in the tress groups.

	Non-TCM *n* = 6,419	TCM *n* = 4,356	Danshen *n* = 525	*p*-value
Age	68.6 ± 12.9	64.2 ± 12.2	63.1 ± 11.4	<0.001
Sex male	5,128 (79.9)	3,031 (69.6)	358 (68.2)	<0.001
CCIS	1.3 ± 1.9	1.2 ± 1.8	1.3 ± 1.9	0.017
Myocardial infarction	19 (0.3)	12 (0.3)	2 (0.4)	0.911
Congestive heart failure	679 (10.6)	385 (8.8)	59 (11.2)	0.007
Peripheral vascular disease	130 (2.0)	98 (2.2)	8 (1.5)	0.473
Cerebrovascular disease	318 (5.0)	162 (3.7)	14 (2.7)	0.001
Dementia	80 (1.2)	29 (0.7)	7 (1.3)	0.010
COPD	685 (10.7)	409 (9.4)	47 (9.0)	0.064
Connective tissue disease	47 (0.7)	56 (1.3)	3 (0.6)	0.009
Peptic ulcer disease	677 (10.5)	590 (13.5)	68 (13.0)	<0.001
Chronic kidney disease	868 (13.5)	546 (12.5)	73 (13.9)	0.289
Hemiplegia	14 (0.2)	5 (0.1)	0 (0)	0.276
Leukemia	5 (0.1)	4 (0.1)	0 (0)	0.778
Malignant lymphoma	24 (0.4)	15 (0.3)	1 (0.2)	0.786
Solid tumor	380 (5.9)	211 (4.8)	33 (6.3)	0.041
Liver disease	277 (4.3)	240 (5.5)	32 (6.1)	0.007
Diabetes mellitus	1,210 (18.9)	766 (17.6)	82 (15.6)	0.072
Hypertension	2,889 (45.0)	1,788 (41.0)	222 (42.3)	<0.001
Event				<0.001
None	5,014 (78.1)	3,654 (83.9)	462 (88.0)	
MACE	638 (9.9)	353 (8.1)	26 (5.0)	
Death	767 (11.9)	349 (8.0)	37 (7.0)	
Event F/u (year)	4.3 ± 3.1	3.8 ± 2.7	4.9 ± 2.8	<0.001
Overall death	935 (14.6)	407 (9.3)	42 (8.0)	<0.001
Overall death F/u (year)	5.0 ± 3.2	4.4 ± 2.7	5.4 ± 2.8	<0.001
First hospitalization	5,249 (81.8)	2,966 (68.1)	368 (70.1)	<0.001
Time to first hospitalization, median (month) (Q1, Q3)	2.7 (0.7, 8.1)	6.5 (1.9, 19.3)	9.9 (2.5, 26.8)	<0.001
Socioeconomic status				<0.001
Low SES	2,232 (34.8)	1,345 (30.9)	163 (31.0)	
Moderate and high SES	4,187 (65.2)	3,011 (69.1)	362 (69.0)	
Geographic region				0.079
Northern/Central	3,791 (59.1)	2,527 (58.0)	285 (54.3)	
Southern/Eastern	2,628 (40.9)	1,829 (42.0)	240 (45.7)	

CCIS, charlson comorbidity index score; COPD, chronic obstructive pulmonary disease; F/u, follow-up; MACE, major adverse cardiac events; Q, quartile; SES, socioeconomic status; TCM, traditional Chinese medicine.

### 3.2 Association between Danshen use and clinical outcomes

**FIGURE 2:**
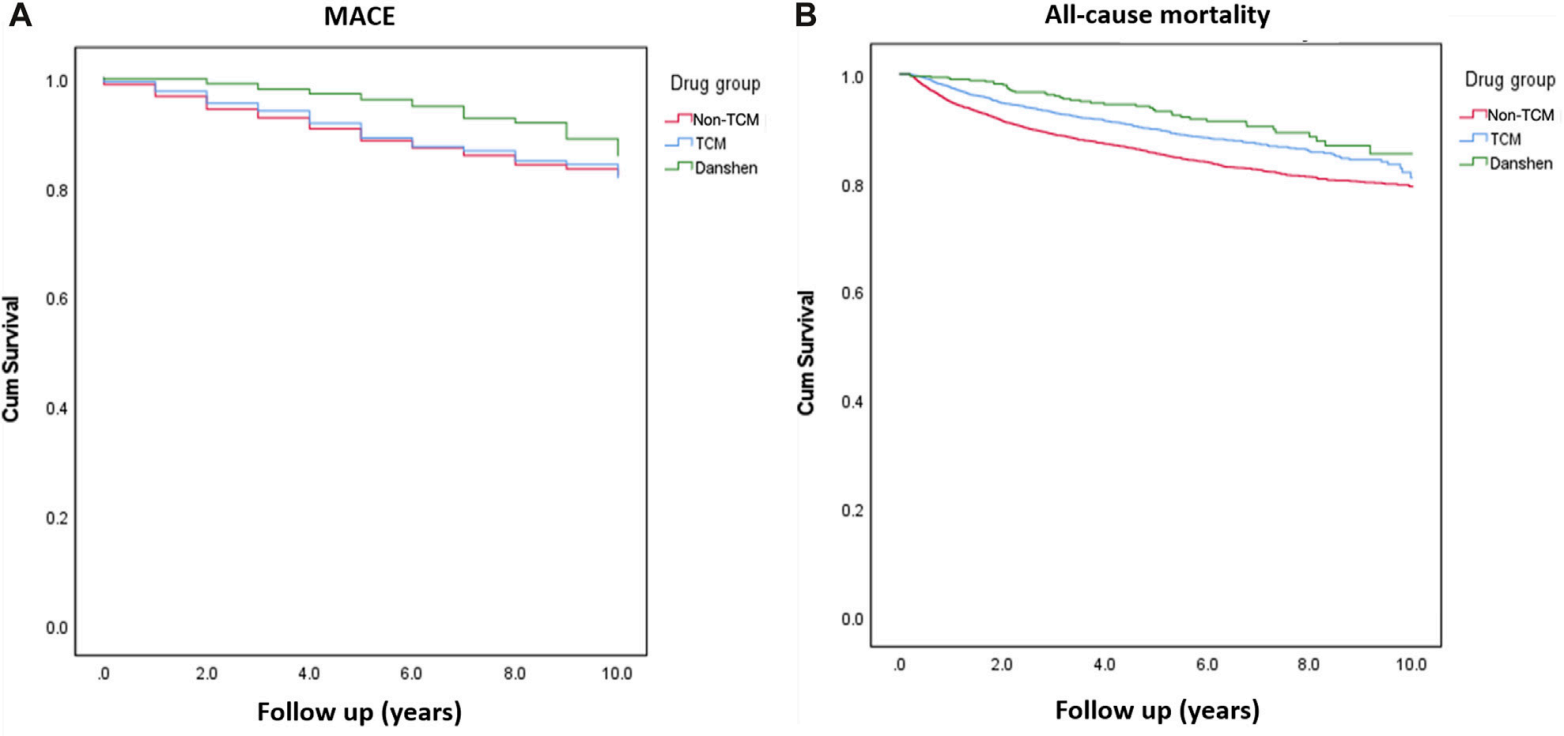
Kaplan–Meier curves of the cumulative incidence of MACE **(A)** and all-cause mortality **(B)** in patients with bladder cancer who were treated with Danshen, traditional Chinese medicine without Danshen (TCM), or therapies other than TCM (non-TCM). The log-rank test was used to compare the Kaplan–Meier curves. Both parameters had significant group differences with *p* < 0.001. MACE, major adverse cardiac events.

The patients in the Danshen group had the lowest crude hazard ratio (HR) for all-cause mortality [HR: 0.51 (0.37–0.69)] and MACE [HR: 0.44 (0.30–0.65)] in the Cox proportional model (Table 2). Age was a significant risk factor for MACE and mortality in the adjusted model [adjusted HR (aHR): 1.04 (1.03–1.04) for MACE and aHR: 1.02 (1.02–1.03) for all-cause mortality]. While sex was a risk factor for MACE in the model, male sex posed a greater risk for all-cause mortality, with an aHR of 1.16 (1.02–1.32). A higher CCIS posed a greater risk for all-cause mortality, with an aHR of 1.17 (1.14–1.2), but not for MACE. Diabetes mellitus, hypertension, chronic obstructive pulmonary disease, and chronic kidney disease were risk factors for MACE but not for all-cause mortality. However, a higher socioeconomic status lowered the risk of all-cause mortality, although not MACE, with an aHR of 0.56 (0.5–0.62). Kaplan–Meier survival curves yielded similar results; the Danshen group showed better survival and lower MACE rates than the other groups (all *p* < 0.001) (Figure 2).

**TABLE 2:** Cox’s proportional hazard model in patients of the Danshen group.

	MACE				Death			
	Crude HR (95%CI)	*p*-value	Adjusted HR (95%CI)	*p*-value	Crude HR (95%CI)	*p*-value	Adjusted HR (95%CI)	*p*-value
Drug Group								
Non-TCM	Ref.		Ref.		Ref.		Ref.	
TCM	0.91 (0.80–1.04)	0.172	1.12 (0.98–1.28)	0.106	0.69 (0.62–0.78)	<.001	0.81 (0.72–0.91)	<.001
Danshen	0.44 (0.30–0.65)	<.001	0.56 (0.38–0.84)	0.004	0.51 (0.37–0.69)	<.001	0.60 (0.44–0.82)	<.001
Age	1.04 (1.03–1.05)	<.001	1.04 (1.03–1.04)	<.001	1.03 (1.02–1.03)	<.001	1.02 (1.02–1.03)	<.001
Male sex	1.14 (0.98–1.33)	0.074			1.16 (1.02–1.31)	0.026	1.16 (1.02–1.32)	0.025
CCIS	1.07 (1.04–1.11)	<.001	0.96 (0.92–1.00)	0.059	1.18 (1.16–1.21)	<.001	1.17 (1.14–1.2)	<.001
Diabetes mellitus	1.76 (1.53–2.02)	<.001	1.50 (1.28–1.74)	<.001	1.29 (1.14–1.47)	<.001	0.99 (0.86–1.13)	0.854
Hypertension	2.10 (1.85–2.38)	<.001	1.56 (1.37–1.78)	<.001	1.34 (1.20–1.49)	<.001	1.02 (0.91–1.14)	0.765
COPD	1.66 (1.39–1.97)	<.001	1.25 (1.04–1.50)	0.019	1.70 (1.47–1.96)	<.001	1.12 (0.96–1.31)	0.138
Chronic kidney disease	1.51 (1.29–1.78)	<.001	1.71 (1.41–2.07)	<.001	1.51 (1.32–1.74)	<.001	1.09 (0.94–1.28)	0.252
Socioeconomic status								
Low SES	Ref.		Ref.		Ref.		Ref.	
Moderate and high SES	0.77 (0.68–0.88)	<.001	0.91 (0.80–1.04)	0.166	0.50 (0.45–0.55)	<.001	0.56 (0.5–0.62)	<.001
Geographic region								
Northern/Central	Ref.				Ref.		Ref.	
Southern/Eastern	1.03 (0.91–1.17)	0.603			0.80 (0.72–0.89)	<.001	0.83 (0.74–0.92)	<.001

CI, confidence interval; CCIS, charlson comorbidity index score; COPD, chronic obstructive pulmonary disease; HR, hazard ratio; MACE, major adverse cardiac events; Ref., reference; SES, socioeconomic status; TCM, traditional Chinese medicine.

## 4 Discussion

In this study, we found that Danshen use was associated with a 40% decrease in mortality and a 44% reduction in MACE compared with those in the TCM and non-TCM groups. Compared to previous studies on Danshen’s effects on cardiovascular outcomes in cancer patients, our findings provide a more comprehensive understanding, especially in the context of bladder cancer. Patients with bladder cancer treated with Danshen showed a significantly lower incidence of MACE and improved survival rates. A meta-analysis of 20 randomized controlled trials including 2,574 patients with coronary heart disease showed that Danshen decreased the MACE risk ratio by 47% (Li et al., 2020). Notably, sodium tanshinone IIA (TSN IIA) sulfonate isolated from Danshen exerted a cardioprotective effect by reducing myocardial infarct size in a rabbit model, suggesting beneficial effects on the clinically crucial vascular endothelium (Wu et al., 1993).

Cardiovascular toxicity caused by cancer treatments has attracted the attention of both cardiologists and oncologists. Chemotherapeutic drugs, cancer-targeting agents, and irradiation can cause cardiovascular damage, leading to plaque formation, thrombosis, arrhythmia, or cardiomyopathy (Weaver et al., 2013). Left ventricular dysfunction and congestive heart failure caused by accumulated anthracycline doses are among the most well-established cardiotoxic side effects (Volkova and Russell, 2011). Emotional stress in patients with cancer may also cause Takotsubo (stress-induced) cardiomyopathy (Haugnes et al., 2010). Moreover, cardiovascular risk factors such as hypertension, diabetes mellitus, obesity, smoking, and inactivity are common among cancer survivors (Weaver et al., 2013). Additionally, epidemiological data have identified similar risk factors, such as smoking, obesity, an imbalanced diet, and insufficient physical exercise, for both cancer and cardiovascular diseases (Johnson et al., 2016). Notably, cardiotoxicity is a known side effect of numerous anticancer treatments that increases the risk of cardiovascular disease in patients with cancer (Weaver et al., 2013). For instance, patients with testicular cancer treated with the chemotherapeutic drug cisplatin, which is frequently used to treat bladder cancer, have an increased risk of coronary artery disease (Huddart et al., 2003; Haugnes et al., 2010). The long-term side effects of cancer therapies such as cisplatin administration also include hypertension (Sagstuen et al., 2005).

TCM offers an alternative method for treating cardiovascular damage caused by cancer treatment. According to the TCM theory, Danshen is highly efficient in promoting circulation, removing blood stasis, and blood thinning (Cheng, 2006). Purified S. miltiorrhiza extracted from Danshen was evaluated for myocardial ischemia/reperfusion injury in isolated rat hearts, and post-ischemic contractile function recovered significantly better in the hearts of the Danshen-treated group (Chang et al., 2006). By preventing the expression of profibrotic molecules, inflammation, and cell death, Danshen dripping pill has been shown to protect against myocardial damage caused by doxorubicin or isoprenaline in C57BL/6 mice in a heart failure model (Feng et al., 2021). Danshen contains the pharmacologically active ingredient TSN IIA, which exerts cardioprotective effects. TSN IIA prevents Leu27 insulin-like growth factor II-enhanced insulin-like growth factor II receptor-mediated cardiac apoptosis (Chou et al., 2022). In the current study, Danshen therapy offered better cardiac protection and decreased the risk of MACEs compared with other TCM and non-TCM treatments in patients with bladder cancer. While our study is based on data from Taiwan, the findings may have implications for broader populations. However, cultural, genetic, or healthcare system factors in Taiwan might influence the results.

Several studies have explored the relationship between Danshen and its effects on cardiovascular and cancer outcomes. Our findings align with some of these studies and provide a unique perspective on bladder cancer patients. In recent years, research on the mechanisms underlying the antitumor properties of active Danshen ingredients has progressed, and Danshen has been used as an anticancer treatment. Studies have identified numerous key anticancer components in Danshen, including di-hydroisotanshinone I (DT), which has been demonstrated to cause cell death. In a rat model of head and neck squamous cell carcinoma, p38 signaling was shown to partially control DT-induced cell death and reduce tumor growth (Hsu et al., 2021). Another study revealed that DT prevents prostate cancer cells from attracting macrophages and inhibits the expression of signal transducer and activator of transcription 3 (STAT3), which causes cancer cells to secrete chemokine ligand 2 (Hsu et al., 2021). Another active Danshen component, cryptotanshinone, influences the Janus kinase-2/STAT3 signaling pathway, causing esophageal cancer cells to undergo apoptosis (Ji et al., 2019). Additionally, cryptotanshinone diminishes the effects of dynamin-related protein 1 and fragments mitochondria (Yen et al., 2019). In addition to safeguarding the cardiovascular system, TSN IIA exhibits anticancer properties. TSN IIA controls the STAT3/chemokine ligand 2 signaling pathway to prevent epithelial-mesenchymal transition in bladder cancer cells (Huang et al., 2017). By encouraging the activation of anti-protein kinase RNA-like endoplasmic reticulum kinase, anti-activating transcription factor 6, caspase-12, caspase-3, inositol-requiring enzyme 1, and anti-CCAAT enhancer-binding protein homologous protein, TSN IIA can cause endoplasmic reticulum stress in pan-cancerous cells (Chiu and Su, 2017).

This study used the NHIRD, which is frequently evaluated for diagnostic accuracy by the NHI Bureau of Taiwan, as a nationwide population database. Approximately 99% of Taiwan’s population and hospitals are represented in the database, which contains all ambulatory and inpatient treatment records (Hsieh et al., 2019). Taiwan also offers equitable access to healthcare for all citizens, creating excellent circumstances for epidemiological research. A recent study based on the Taiwan NHIRD found that the highest usage rate of Danshen was 9.48% for menstrual disorders and abnormal bleeding from the female genital tract, whereas usage for cardiovascular symptoms was the third most common application at 4.18% (Tseng et al., 2021). After considering comorbidities, our population-based study found that Danshen dramatically reduced the incidence of MACEs in patients with bladder cancer. Moreover, the mortality rate was lower in the Danshen group than in the non-TCM group. Building on our findings, a prospective study or randomized controlled trial would be a valuable next step. Investigating the relationship between Danshen and cardiovascular outcomes in other populations or settings would also be beneficial.

### 4.1 Limitations

This study had some limitations, including potential biases due to its retrospective nature. Limitations in data sources and the categorization of variables might also influence the results., in which two or more experts reviewed the data upon confirmation of the diagnosis, we were unable to fully verify these data. While we adjusted for several potential confounders in our analysis, we acknowledge that information on certain confounders, like alcohol consumption and smoking status, is not included in the NHIRD. Additionally, it is crucial to note that we are unable to ascertain the specific staging of bladder cancer in this study. This lack of granularity in the data may mask potential variances in the effects of Danshen across different cancer stages. In future research, a more detailed, stage-specific analysis would indeed be beneficial to provide more nuanced insights. Future research would benefit from a more detailed, stage-specific analysis to provide nuanced insights. Further studies are also warranted to explore the mechanism of action of Danshen in patients with bladder cancer.

An additional limitation to note is that TCM treatments are typically personalized, based on individual patient conditions. Due to the limitations of the NHIRD, detailed consultation data, including specific treatments for various reasons, is not available. Nonetheless, we have endeavored to control for potential variability in complex conditions using Cox’s proportional hazard model in Table 2. Regarding conventional drug treatments for the non-TCM group, the NHIRD does not provide specific details about the medications prescribed to the patients in our study.

## 5 Conclusion

Danshen may be useful in reducing the risks of MACE and all-cause mortality in patients with bladder cancer. The observed benefits suggest its potential integration into standard care protocols, especially for patients at risk of cardiovascular events. In conclusion, our study highlights the potential benefits of Danshen in reducing cardiovascular risks in bladder cancer patients. These findings pave the way for further research and potential clinical applications.

## Data Availability

The original contributions presented in the study are included in the article/Supplementary Material, further inquiries can be directed to the corresponding author.

## Abbreviations

aHR, adjusted hazard ratio; CCIS, Charlson Comorbidity Index Score; CCL2, C-C motif ligand 2; DT, di-hydroisotanshinone I; HR, hazard ratio; ICD-9, International Classification of Diseases, ninth revision; MACE, major adverse cardiac events; NHI, National Health Insurance; NHIRD, National Health Insurance Research Database; STAT3, signal transducer and activator of transcription 3; TCM, traditional Chinese medicine; TSN IIA, sodium tanshinone IIA.
